# The Effects of Different Roasting Methods on the Phenolic Contents, Antioxidant Potential, and In Vitro Inhibitory Activities of Sacha Inchi Seeds

**DOI:** 10.3390/foods12224178

**Published:** 2023-11-20

**Authors:** Suwapat Kittibunchakul, Varongsiri Kemsawasd, Chatrapa Hudthagosol, Promluck Sanporkha, Suwimol Sapwarobol, Uthaiwan Suttisansanee

**Affiliations:** 1 Food and Nutrition Academic and Research Cluster, Institute of Nutrition, Mahidol University, Salaya, Phuttamonthon, Nakhon Pathom 73170, Thailand; suwapat.kit@mahidol.ac.th (S.K.); varongsiri.kem@mahidol.ac.th (V.K.); 2 Faculty of Public Health, Mahidol University, Ratchathewi, Bangkok 10400, Thailand; chatrapa.hud@mahidol.ac.th (C.H.); promluck.san@mahidol.ac.th (P.S.); 3 Faculty of Allied Health Sciences, Chulalongkorn University, Pathumwan, Bangkok 10330, Thailand; suwimol.sa@chula.ac.th

**Keywords:** *Plukenetia volubilis*, bioactive compounds, antioxidant activity, enzyme inhibition, glycation, non-communicable diseases

## Abstract

Roasted sacha inchi seeds are now commercialized as a health food product, but the influence of roasting methods on their proclaimed health effects has yet to be explored. This study investigated the total phenolic contents (TPCs), antioxidant potential, and inhibitory activities of raw and roasted sacha inchi seeds in vitro. Individual phenolics in raw seeds were also identified in an attempt to explain the bioactivities of the seeds. The results suggested that roasting in a cooking pan, vacuum oven, and tray dryer had distinct impact on TPC in sacha inchi seeds, and thus differentially altered their antioxidant and inhibitory properties. Seeds that underwent roasting exhibited 1.5–2.7-fold higher antioxidant potentials than raw seeds. Certain roasting methods provided the products with anti-α-amylase and anti-cholinesterase activities, while inhibitions of these enzymes were not detected in raw seeds. Roasted seeds also possessed superior anti-lipase and anti-glycation activities when compared with raw seeds (up to 1.7- and 4.8-fold, respectively). The inhibitory properties observed in the seed samples might be attributed to their *p*-coumaric acid, ferulic acid, and quercetin, as these potential enzyme inhibitors were predominant in raw seeds. The overall results showed that pan-roasting could be used to obtain relatively high health benefits from the antioxidant and inhibitory activities of sacha inchi seeds. The information obtained from this study may serve as the basis for the proper processing of sacha inchi seeds to optimize their functional food and nutraceutical applications.

## 1. Introduction

Sacha inchi (*Plukenetia volubilis* L.) or the Inca peanut is an important indigenous food crop in Latin America, and also a traditional remedy employed by local communities in the Amazon region [1,2]. The plant produces an oleaginous seed kernel as the main edible part, possessing an excellent nutritional profile with high levels of protein (25–30%, including essential amino acids) and lipids (35–60%, comprising mainly polyunsaturated fatty acids) [3,4]. Sacha inchi seeds are also rich in bioactive phytochemicals such as polyphenols, carotenoids, tocopherols and phytosterols [1,5,6], making them highly attractive for dietary use. Recently, sacha inchi production has increased in many tropical countries, particularly in South America and Southeast Asia, where the plant contributes greatly to economic development [7].

The health benefits associated with sacha inchi seeds have been supported by scientific research. Previous studies have demonstrated that sacha inchi seeds, as well as their extracted oil, exhibit high antioxidant activities and had suppressive effects on non-communicable disorders (NCDs) such as coronary heart disease and hypertension [1,8]. Positive effects related to the consumption of sacha inchi seeds were confirmed in vivo. A clinical study reported that the daily intake of 30 g of seeds for 6 weeks significantly improved the serum lipid profile in adults [9], while another clinical study on the biological activity of sacha inchi oil suggested that long-term intake of the oil reduced total serum cholesterol and non-esterified fatty acid levels with high-density lipoprotein cholesterol (HDL-C) elevation in hypercholesterolemia patients [8]. Sacha inchi oil also exhibited anti-cancer effects in tumor-bearing rats by reducing the proliferation of tumor cells. Reductions in blood sugar, triglycerides, and inflammatory cytokine levels were also observed after treating the rats with sacha inchi oil [10], promoting sacha inchi seeds as a functional food.

Traditionally, sacha inchi seeds are consumed as a gourmet oil or cooked by pan-roasting, steaming, boiling, and other conventional cooking methods [11]. The processing of sacha inchi seeds has been gaining attention from the food industry for commercial purposes over the past decade. Currently, most seeds are subjected to a pressing process and sold as a high-quality edible oil [12]. Whole seeds are also commercialized as savory snacks after being subjected to industrial cooking operations like roasting in a large-scale oven or dryer. However, high temperatures during cooking processes can diminish the nutritional quality and health properties of sacha inchi seeds [11]. Previous studies have suggested that roasting and boiling alters fatty acid profiles and mineral contents in sacha inchi seeds, with roasting being superior to boiling in terms of preserving seed nutritional components and physicochemical characteristics [11,13]. Several studies have focused on the nutritive value, phytochemical composition and health-promoting effects of sacha inchi seeds, but the influence of thermal processing on seed health properties has not been extensively explored.

This comparative study investigated the phenolic content, antioxidant activities, and inhibitory properties of sacha inchi seeds both raw and cooked using different roasting methods. Cooked seeds were prepared by roasting in a cooking pan, vacuum oven or tray dryer to mimic the processes generally used for producing snacks from whole nuts. Enzyme inhibitory assays were conducted using the key enzymes associated with common metabolic (type II diabetes, obesity and hypertension) and neurodegenerative (Alzheimer’s disease) disorders. Inhibition of non-enzymatic glycation was also observed in vitro. Furthermore, we identified phenolic compounds in raw sacha inchi seeds in an effort to explain the health properties of the seeds. The results obtained from the present study can support further research on the bioactivities of roasted sacha inchi seeds and also serve as the basis for the proper processing of sacha inchi seeds for functional food and nutraceutical applications.

## 2. Materials and Methods

### 2.1. Sample Preparation and Extraction

Sacha inchi seeds, with kernels separated from shells, were kindly provided by Thai Rubber Land and Plantation Co., Ltd., Muang, Chiang Rai, Thailand. We conducted all experiments within one year after obtaining the samples in March 2014. The proximate composition and appearance of the seeds are presented in Supplementary Table S1 and Figure S1, respectively. Whole unbroken seeds were selected, homogenized and divided into four groups of 100 g each. The first was used for analysis of the raw sample. The second was roasted in a cooking pan at ~100 °C for 20 min. The third was roasted in a vacuum oven (Model VD53, Binder GmbH, Tuttlingen, Germany) at 150 ℃ for 20 min, and the fourth was roasted in a hot-air tray dryer (Model King 0.37 kw/0.5 HP, King Kluaynamthai, Bangkok, Thailand) at 150 ℃ for 20 min. The samples were ground into powder (particle size < 0.5 mm) using a high-speed miller (Model JPS-500, Yongkang Horus Industry and Trade, Yongkang, Zhejiang, China). Moisture contents and color analyses of the powdery samples are provided in Supplementary Table S2. All samples were kept frozen in vacuum foil bags at −20 °C until used.

Extraction of sacha inchi samples was conducted following Kittibunchakul et al. (2022) [12]. Briefly, 0.2 g of the powdery samples were extracted with 100 mL of 20% (*v*/*v*) aqueous ethanol in a 70 °C water bath shaker for 15 min. The supernatants were received after centrifugal solid–liquid separation (3800× *g*, 10 min) and then filtered using 0.45 µM PES membranes. The clear extracts were collected and kept at −20 °C until analysis. For further investigations, all reagents and chemicals were obtained from Sigma-Aldrich (St. Louis, MO, USA), unless stated otherwise, and were of the highest quality.

### 2.2. Determination of Phenolic Profile and Total Phenolic Content

Analysis of the phenolic profiles was performed following the method of Temviriyanukul et al. (2020) with no modifications [14]. Briefly, raw seed samples were extracted with acidic methanol before injecting the extract into the high-performance liquid chromatography (HPLC) system (with previously explained conditions and a gradient elution process). The HPLC chromatogram was collected using the wavelength detections for phenolic acid detection at 280 and 325 nm, with flavonoid detection at 338 and 368 nm.

Total phenolic contents (TPCs) were determined using a microplate reader (Model Synergy^TM^ HT, BioTek Instruments Inc., Winooski, VT, USA) with BioTek Gen5 software (version 2.09), as previously described by Hinkaew et al. (2020) with no modifications [15]. In brief, the extracted sample was mixed with 10% (*v*/*v*) Folin–Ciocalteu phenol reagent and 7.5% (*w*/*v*) sodium carbonate at 25 °C for 2 h. Subsequently, the absorbance value was determined as an end-pointed assay at 765 nm, and results were reported as mg gallic acid equivalent (GAE)/100 g dry weight (DW).

### 2.3. Determination of Antioxidant Activity

Antioxidant activities were determined spectrophotometrically with a microplate reader utilizing the ORAC, FRAP and DPPH radical scavenging assays. All assays followed protocols previously described by Sripum et al. (2017) with no modifications [16]. Briefly, the ORAC assay was performed using the extracted sample and AAPH solution. Activity was observed as a fluorescence decay kinetics curve of fluorescein at an excitation wavelength (λ_ex_) and emission wavelength (λ_em_) of 485 nm and 528 nm, respectively. The FRAP assay was conducted by incubating the extracted sample with the FRAP reagent at 25 °C for 8 min, and the absorbance was subsequently measured as an end-pointed assay at 600 nm. The DPPH radical scavenging assay was conducted by incubating the extracted sample with DPPH reagent at 25 °C for 30 min before the absorbance measurement as an end-pointed assay at 520 nm. For all the antioxidant activity assays, results were reported as µmol Trolox equivalent (TE)/100 g DW.

### 2.4. Determination of Enzyme Inhibitory Activity

Inhibitions of the key enzymes associated with type II diabetes (α-amylase and α-glucosidase), obesity (lipase), and Alzheimer’s disease (acetylcholinesterase; AChE, butyrylcholinesterase; BChE, and β-secretase 1; BACE-1) were investigated in vitro following protocols previously described by Sirichai et al. (2022) with no modifications [17]. The inhibition of hypertension-associated enzyme (angiotensin converting enzyme; ACE) was observed following the report of Chupeerach et al. (2021) [18]. Components of all enzymatic reactions are shown in Table 1. The reactions were visualized on the microplate reader at particular wavelengths. Enzyme inhibitory activities were calculated as percentages of inhibition at a particular extract concentration using Equation (1).

(1) $$\%\;\mathrm{Inhibition}\;=\;\left(1-\frac{\left(B-b\right)}{\left(A-a\right)}\right)\times 100$$

where *A* is the initial reaction rate of the control reaction with enzyme but without the sample extract, *a* is the initial reaction rate of the control reaction without enzyme and the sample extract, *B* is the initial reaction rate of the enzymatic reaction with the sample, and *b* is the initial reaction rate of the reaction with the sample but without enzyme.

### 2.5. Determination of Glycation Inhibitory Activity

The inhibition of non-enzymatic protein glycation of bovine serum albumin (BSA) was investigated, as previously described by Kittibunchakul et al. (2022), with no modifications [12]. In brief, the extracted sample was mixed with BSA and either methylglyoxal (MG) or D-glucose, and then incubated at 37 °C for 21 days. Subsequently, advanced glycation end products (AGEs)’ formation was detected on the microplate reader as an end-pointed assay at λ_ex_ 330 nm and λ_em_ 410 nm. The inhibitory activities reported as percentages of inhibition at a particular extract concentration were calculated using Equation (1).

### 2.6. Statistical Analysis

All experiments were conducted in triplicate (*n* = 3), with results presented as mean ± standard deviation (SD). An ANOVA followed by Duncan’s multiple range test was used to compare significant differences between mean values. An unpaired *t*-test was used to compare significant differences between the values of two data sets. All statistical tests were conducted using SPSS software (version 23.0, SPSS Inc., Chicago, IL, USA), and values were considered significantly different at *p*-values less than 0.05 (*p* < 0.05).

## 3. Results

### 3.1. The Effect of Roasting Processes on Total Phenolic Content

**Figure 1:**
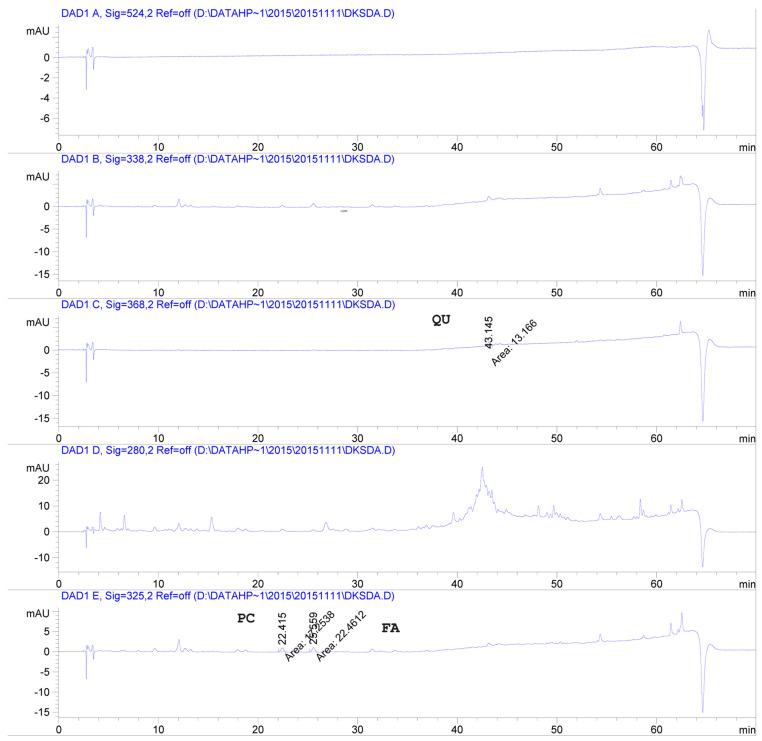
An example of HPLC chromatograms of individual phenolics identified in raw sacha inchi seeds. Peaks were identified with retention times (t_R_) compared with authentic standards, and confirmed the characteristic spectra by photodiode array. QU: quercetin (t_R_ = 43.15 ± 0.27 min); PC: *p*-coumaric acid (t_R_ = 22.42 ± 0.29 min); FA: ferulic acid (t_R_ = 25.56 ± 0.33 min).

The major phenolic compounds in our raw seeds were identified as *p*-coumaric acid (84.38 µg/100 g DW), ferulic acid (90.26 µg/100 g DW), and quercetin (71.65 µg/100 g DW) by HPLC analysis compared with 17 authentic phenolic standards, including 8 flavonoids (myricetin, quercetin, luteolin, naringenin, hesperitin, kaempferol, apigenin and isorhamnitin) and 9 phenolic acids (gallic acid, 4-hydroxybenzoic acid, chlorogenic acid, vanillic acid, caffeic acid, syringic acid, *p*-coumaric acid, ferulic acid and sinapic acid) (Table 2 and Figure 1). As shown in Table 3, the Folin–Ciocalteu assay revealed that roasting quantitatively affected the phenolic compounds in sacha inchi seeds. Raw sacha inchi seeds possessed a TPC of 45.15 mg GAE/100 g DW, which significantly increased 1.2–1.3-fold after roasting in a heated pan and a vacuum oven. By contrast, roasting in a hot-air tray dryer had a negative impact, after which TPC significantly decreased by 1.1-fold compared to raw seeds.

### 3.2. The Effect of Roasting Processes on Antioxidant Activity

The capacity to resist oxidation was explored in vitro using ORAC, FRAP and DPPH radical scavenging assays, with results shown in Table 3. Roasting sacha inchi seeds under the studied conditions resulted in a significant increase in antioxidant activities. Seeds cooked by pan-roasting exhibited the highest ORAC and FRAP values (284.48 and 4.27 µmol TE/100 g DW, respectively), followed by seeds roasted using a tray dryer (251.92 and 4.07 µmol TE/100 g DW, respectively) and vacuum oven (161.42 and 3.68 µmol TE/100 g DW, respectively). These ORAC and FRAP values were 1.5–2.7-fold and 1.5–1.7-fold higher than the ORAC and FRAP values obtained from raw seeds (106.14 and 2.47 µmol TE/100 g DW, respectively). Similar results were observed for DPPH radical scavenging activities. The DPPH radical scavenging values of the pan-roasted and tray dryer-roasted seeds were comparable (0.015–0.018 µmol TE/100 g DW) and significantly 1.3–1.5-fold higher than those of oven-roasted seeds and raw seeds (0.012–0.014 µmol TE/100 g DW).

### 3.3. The Effect of Roasting Processes on Enzyme Inhibitory Activity

The health-related properties of raw and roasted seeds of sacha inchi were explored in vitro by determining the inhibition of the key enzymes relating to type II diabetes (α-amylase and α-glucosidase), obesity (lipase), hypertension (ACE), and Alzheimer’s disease (AChE, BChE and BACE-1). Results were presented as percentages of inhibition at a final extract concentration of 0.4 mg of sacha inchi seeds per mL of 20% (*v*/*v*) aqueous ethanol for comparison, as shown in Table 4.

Inhibition of the carbohydrate-hydrolyzing enzymes, α-amylase and α-glucosidase, retarded starch degradation, slowed down the release of glucose, and thus reduced the glucose absorption and blood glucose levels. Therefore, this was adopted as a diabetes prevention strategy. The results indicated that only α-amylase inhibitory activity was detected using an extract concentration of 0.4 mg/mL, while the α-glucosidase inhibitory activity of all the samples was undetected using the same extract concentration. However, α-amylase inhibitory activity was only detected in sacha inchi seeds roasted using a vacuum oven (16.33% inhibition), with no activity observed in the other samples. The half-maximal inhibitory concentration (IC_50_) of vacuum oven-roasted seeds on this enzyme was, thus, higher than 0.4 mg/mL.

Inhibition of the lipid-degrading enzyme, lipase, suppresses obesity by reducing fat absorption in the body. The results showed that all sacha inchi seed samples retarded lipase activity by 14.63–24.19% using an extract concentration of 0.4 mg/mL, with IC_50_ values determined to be higher than 0.4 mg/mL. The highest lipase inhibitory potential was found in vacuum oven-roasted seeds, being 1.2-, 1.4- and 1.7-fold higher than the lipase inhibitory activities of tray dryer-roasted, pan-roasted, and raw seeds, respectively.

Inhibition of ACE lowers angiotensin II, a signaling peptide that elevates blood pressure and promotes hypertension. In this study, no ACE inhibitory activity was observed in all sacha inchi seed samples using an extract concentration of 0.4 mg/mL.

A decrease in acetylcholine via the activity of AChE and BChE is an important biomarker in the diagnosis of Alzheimer’s disease. Apart from the cholinergic system, the deposition of misfolded amyloid-beta (Aβ) in the brain is also a hallmark of this disease. The formation of Aβ plaques is induced by the hydrolysis of amyloid precursor protein by BACE-1. BACE-1 inhibition has thus been shown to be a therapeutic approach to this neurodegenerative disorder. Among all studied sacha inchi seed samples, AChE inhibitory activity was only observed in seeds cooked via pan-roasting (12.25% inhibition) using an extract concentration of 0.4 mg/mL, indicating that its IC_50_ was higher than 0.4 mg/mL. BChE inhibition was also observed at the same extract concentration, with inhibitory activities only observed in pan-roasted and vacuum oven-roasted seeds, while no BChE inhibitory activities were detected in raw seeds and tray dryer-roasted seeds. Interestingly, the pan-roasted seeds exhibited 5.0-fold higher BChE inhibitory activity than that the vacuum oven-roasted seeds, with no BACE-1 inhibitory activity observed in all seed samples using an extract concentration of 0.4 mg/mL.

### 3.4. Effect of Roasting Processes on Glycation Inhibitory Activities

In vitro inhibitions of non-enzymatic glycations by raw and roasted seeds of sacha inchi are reported in Table 5. Inhibitions of glycations by the extracted samples ranged between 15.18–72.66% and 19.40–75.99% of BSA induced by MG and D-glucose, respectively, using a final extract concentration of 0.125 mg/mL. Thus, the IC_50_ of the extracted samples was considered to be lower than 0.125 mg/mL. The inhibitory activities working against MG- and D-glucose-induced glycations followed the same trend, with seeds cooked via pan-roasting possessing the highest inhibitory activities, followed by seeds roasted using a vacuum oven and tray dyer, respectively; raw seeds exhibited the lowest value. Increases in inhibitory activities by 4.4–4.8-fold and 3.9–3.5-fold after roasting processes were observed in anti-glycation reactions induced by MG and D-glucose, respectively. Moreover, significantly stronger inhibitions in the reaction induced by D-glucose than MG were observed in raw seeds and pan-roasted seeds.

## 4. Discussion

Sacha inchi seeds have gained increasing attention from both consumers and the industrial sector due to their attractive nutritional and functional properties. Products made from the seeds such as seasoned seeds, crunchy snacks, protein powder, and omega-3 rich oil supplements are now industrialized [2]. Previous research has identified sacha inchi seeds as a good source of several bioactive substances that modulate physiological functions and ameliorate some NCDs such as oxidative stress-related diseases and glycation reactions [2,7]. Sacha inchi seeds are typically consumed after thermal treatment during culinary preparation or food processing, and the sensory characteristics, chemical composition, and nutritive value of the seeds are often altered [11,13,19]. Distinct cooking processes diversely influence the nutritional and physicochemical properties of sacha inchi seeds [11]. Thermal processes have also been reported to affect phenolic compounds and tocopherols in sacha inchi seeds, leading to the alteration of their antioxidant activities [13]. Copious information on the health properties of sacha inchi seeds is currently available, but scant research has focused on the impacts of thermal processing on the proclaimed health effects of the seeds. Therefore, here, we investigated and compared their TPCs, antioxidant potentials and inhibitory activities against disease-associated enzymes, as well as the non-enzymatic glycation reactions of raw and cooked sacha inchi seeds. The utilized cooking methods including pan-roasting, vacuum oven-roasting, and tray dryer-roasting mimicked the processes used for preparing snacks from whole nuts at both household and industrial levels. The conditions selected also mimicked those of the processes used to prepare snacks from whole nuts. Vacuum oven-roasting and tray dryer-roasting were conducted at 150 °C, which is the temperature recommended for roasting oleaginous nuts/seeds to avoid the formation of carcinogens [20,21]. Pan-roasting at a temperature as high as 150 °C could burn the seeds from outside; therefore, we conducted pan-roasting at around 100 °C.

The phenolic content (45.15 mg GAE/100 g DW) found in our raw sacha inchi seeds was different from the TPC values reported by Chirinos et al. (2013) (65–80 mg GAE/100 g) [1] and Štěrbová et al. (2017) (17 mg GAE/100 g) [13]. Generally, plant phenolics are diverse in terms of structure, molecular size and polarity; hence, extraction systems and analytical techniques can greatly influence the measurement results. In comparison to sacha inchi seed extract previously obtained with 70% acetone [1], the aqueous ethanolic extract observed in this study showed substantially lower TPC. This finding suggested that phenolic compounds in sacha inchi seeds tend to have a low-polarity nature, and further optimization of extraction process (e.g., solvent, temperature and time) is crucial for accurate quantification and identification of phenolics in the seeds. As identified by HPLC analysis, raw seeds contained predominantly *p*-coumaric acid, ferulic acid, and quercetin, which were somewhat distinct from the individual phenolics detected in sacha inchi oil [5], husk, and shell [12]. Besides, considering this phenolic profile in comparison to TPCs, it is possible that phenolics in sacha inchi seed might be other phenolics than the ones used as phenolic standards in our HPLC analysis. The results from this study indicated that roasting had multiple effects on TPC in sacha inchi seeds depending on the roasting method used, concurring with earlier studies reporting the changes in TPCs during thermal treatments of the same parts of sacha inchi [13,19]. When the comparison was made with raw seeds, pan-roasting and vacuum oven-roasting resulted in significant increases in TPCs. Similar findings on the increase in TPCs after the processing of sacha inchi seeds at high temperatures (100–190 °C) were reported by Štěrbová et al. (2017) [13], who suggested that the increase was related to the formation of new compounds harboring phenol moieties. Several studies have also demonstrated that thermal processing effectively increases TPC in foods. Heat causes the breakdown of covalent bonds between phenolic compounds and the food matrix, leading to the improved extraction efficiency of phenolics [18,19]. However, phenolic compounds are often degraded after prolonged exposure to extreme heat [22], explaining the higher TPC observed in our pan-roasted seeds at 100 °C compared with vacuum oven-roasted seeds at 150 ℃, using the same roasting time of 20 min. Among all the sacha inchi seed samples, the tray dryer-roasted seeds exhibited the lowest TPC at 10% lower than TPC in raw seeds. Likewise, roasting in the tray dryer yielded a cooked product with lower TPC compared with roasting in the vacuum oven regardless of temperature and roasting time. A vacuum oven operates by reducing pressure within the oven chamber. This creates an oxygen-free condition, lowers the boiling point, and minimizes the loss of heat-labile compounds [23]. The significant TPC reduction in tray dryer-roasted seeds possibly resulted from the oxidation of some phenolics due to exposure to oxygen and high temperature. Our results agreed well with a previous study that reported that coffee pulp dried using a vacuum oven had a higher TPC content than pulp dried using a hot-air dryer [24].

Thermal processes can alter the natural antioxidant potential of plant foods, and therefore the influence of such processes should be taken into account for accurate evaluation of the beneficial effects of food products [25]. Phenolic compounds are well-recognized as major antioxidants in plants. In our previous study, an intensive correlation between TPCs and antioxidant potentials that was dependent on the extraction procedures was presented [17]. In this study, however, the antioxidant activities of sacha inchi seed samples did not fully agree with the TPCs. Besides phenolics, existing documents reported the presence of non-phenolic antioxidants such as polyunsaturated fatty acids, carotenoids, tocopherols, and phytosterols in sacha inchi seeds [1,5,6] as a plausible explanation for the discrepancy between our current and previous findings. The ORAC, FRAP, and DPPH radical scavenging values indicated that processing at high temperatures helped to enhance the antioxidant activities of sacha inchi seeds, possibly due to the development of non-phenolic antioxidants and/or Maillard reaction products [26,27]. Roasting usually results in the thermal degradation of some naturally occurring phytochemicals in plant materials, but it is also capable of generating antioxidant compounds through the Maillard reaction [28]. Such a phenomenon concerning the positive impacts of thermal processes on antioxidant activities was observed during the roasting of almond and pine nuts [29], concurring with our results. As suggested by the antioxidant activity assays, seeds cooked by pan-roasting were likely to possess the highest antioxidant activities, followed by seeds roasted using a vacuum oven and tray dyer, respectively; hence, pan-roasting could be a promising cooking method of obtaining antioxidants from seeds of sacha inchi. However, this presumption might only be valid for the studied roasting conditions, and different thermal processes might render diverse outcomes.

Control of disease-associated enzymes has recently emerged as a strategy to reduce the incidence of NCDs, enabling the application of targeted enzyme inhibition for drug development in specific disease contexts. This is the first paper to report the effects of roasting on the enzyme inhibitory activities of sacha inchi seeds. Here, we investigated in vitro inhibitory activities against the key enzymes that control some NCDs by using extracts of raw and roasted seeds of sacha inchi. Among the tested enzyme inhibitory assays, inhibitions of α-amylase, lipase, AChE, and BChE by particular extracted samples were detected at an extract concentration of 0.4 mg/mL. The enzyme inhibitory activities of plant-based foods were attributed to their phenolics, because several phenolic compounds in plants play important roles as enzyme inhibitors [25]. Phenolics including *p*-coumaric acid, ferulic acid and quercetin detected in our raw sacha inchi seeds could act as α-amylase inhibitors with IC_50_ values of >30, 9.5 and 2.5 mM [30], respectively. However, inhibitory activities against this diabetes-related enzyme were not present in all the sacha inchi extracts. Only the extract derived from vacuum oven-roasted seeds exhibited this inhibitory activity, suggesting that the quantity of these phenolics extracted from vacuum oven-roasted seeds might be high enough to inhibit α-amylase, while the others might be too low. Cholinesterases including AChE and BChE, the enzymes involved in Alzheimer’s disease, were also inhibited by *p*-coumaric acid and ferulic acid, with inhibitory activities against BChE superior to AChE [31]. Quercetin was also shown to inhibit AChE and BChE successfully, both having IC_50_ values of around 0.2 mM [32]. The mild AChE and BChE inhibitory activities detected in the extracts of pan-roasted and vacuum oven-roasted seeds agreed well with observations for TPCs, as discussed above. No AChE and BChE inhibitory activities were observed in extracts obtained from raw seeds and some roasted samples. The absence of AChE and BChE inhibitions might be because the concentrations of inhibitory compounds in the tested extracts were not high enough to produce the cholinesterase inhibitory effects. Despite their low concentration (0.4 mg/mL), all the extracted samples showed anti-obesity properties through the inhibition of lipase. Most phenolic compounds can act as lipase inhibitors [33]. In particular, *p*-coumaric acid, ferulic acid and quercetin were previously reported to be strong inhibitors for this lipid-degrading enzyme (IC_50_ values of 170.2, 123.9 and 231.6 µM [34], respectively). However, a different trend between TPCs and lipase inhibitory activities was observed in this study, suggesting that apart from the detected phenolics, lipase inhibitors in the samples might include other phenolics (not included in our standards) and non-phenolic substances that were formerly identified as strong anti-lipase agents [35]. The inhibition of enzymes by phytochemicals depends greatly on the chemical compositions of the plant origin, as well as the structural characteristics of inhibitor candidates responsible for the relevant inhibitory effects [25,31]. Metal ions and macromolecules (e.g., polysaccharides and proteins) in crude plant extracts often interact with enzyme inhibitors, leading to the modification of their inhibition efficacies. Synergistic and/or antagonistic effects among phytochemicals may also occur, thereby resulting in changes in the inhibitory potential [31].

A correlation among phenolic compounds, antioxidant activities and glycation inhibitory activities of plant materials was previously reported [17,36]. The finding of this study suggested that roasting of sacha inchi seeds remarkably improved inhibition toward D-glucose- and MG-mediated glycation reactions, concurring with results obtained from the antioxidant activity assays. The extracts of roasted samples had strong anti-glycation activities, indicating that these extracts could alleviate aging process and other disorders linked to non-enzymatic glycation. The anti-glycation capacity of our sacha inchi seed samples was probably related to their phenolic compositions. Phenolics usually inhibit the formation of AGEs in the early glycation phase, but some retard the advanced stage of glycation and also the subsequent inter-protein cross-linking [37]. As predominant phenolics detected in raw seeds in our study, *p*-coumaric, ferulic acid, and quercetin were previously reported to lower AGE formation effectively [38,39,40]. Limited information describes the impacts of processing at high temperatures on the anti-glycation effect, but a recent study on plant phenolics reported that phenolic compounds under thermal conditions exhibited enhanced inhibitory effects against glycation [41]. The study also underlined the mechanisms whereby high-temperature treatment led to a decrease in AGE levels and the reduction of electrophiles formed by an in vitro digestion system [41]. Given their glycation inhibitory activities, roasted sacha inchi seeds, especially those prepared by pan-roasting, were shown to be potentially important sources of glycation inhibitors.

Overall, our results suggest that pan-roasting under the studied conditions could be used to obtain relatively high health benefits from the antioxidant and inhibitory activities of sacha inchi seeds. Pan-roasting relies primarily on heat conduction. The direct conductive transfer of thermal energy between a moderately heated object and the low-moisture sample seemed to promote the formation of Maillard reaction-derived antioxidants and certain enzyme inhibitors more effectively than cooking techniques with other heat transfer mechanisms such as boiling (convection) and microwaving (radiation), concurring with the study of Liao et al. (2012) [42]. On the contrary, during vacuum oven-roasting, the Maillard reaction and any chemical modifications are avoided due to the anoxic atmosphere and the presence of inert gases [43]. Besides, previous research supports the present study’s finding that hot-air drying strongly induced lipid oxidation, leading to the degradation of phenolic antioxidants and lipid-based functional molecules [24,44]. This could be a likely explanation for the better in vitro health properties observed for the pan-roasted seeds.

## 5. Conclusions

Sacha inchi seeds are nutritious and contain several phytochemicals with potential health-promoting effects. The seeds are usually processed into food products by roasting in a pan, as well as in an industrial oven or dryer for commercial purposes. Roasting affects the chemical composition of sacha inchi seeds, which in turn influences their TPC, antioxidant potentials, and in vitro inhibitory activities. The effects of roasting on the seeds were greatly dependent on the roasting method used. Roasting under the studied conditions potentially results in cooked seeds with improved health properties. The inhibitions of disease-associated enzymes and glycation reactions by the seed extracts suggested that phenolic compounds might be responsible for some of these inhibitory effects; however, other phytochemicals (e.g., carotenoids, tocopherols, phytosterols and fatty acids) might also play roles in these inhibitions. In this study, inhibitory activities were only reported using a particular extract concentration, making a comparison of our results in enzyme inhibitions rather limited. In addition, further optimization of the extraction process is still required for a detailed phytochemical analysis of sacha inchi seeds. Nevertheless, the information obtained from the present study can support further research on the health properties (including bioaccessibility and toxicity) of roasted sacha inchi seeds using human cell line models or in vivo investigations. Knowledge gained from this research may serve as the basis for the proper processing of sacha inchi seeds for optimal functional food and nutraceutical applications, using thermal conditions that maximize the retention of health-related activities.

## Figures and Tables

**Table 1:** Components of the enzymatic assays.

Assay	Enzyme	Substrate	Indicator	Detected Wavelength
α-Amylase	≥10 units/mg (type VII), porcine pancreatin	*p*-nitrophenyl-α-D-maltopentaoside		405 nm
α-Glucosidase	≥10 units/mg (type I), *Saccharomyces cerevisiae*	*p*-nitrophenyl-α-D-glucopyranoside		
Lipase	≥700 units/mg (type VII), *Candida rugose*	DMPTB	DTNB	412 nm
AChE	1000 units/mg, *Electrophorus electricus*	acetylthiocholine		
BChE	≥10 units/mg, equine serum	butyrylthiocholine		
BACE-1	BACE-1 FRET assay kit (Sigma-Aldrich, St. Louis, MO, USA)			λ_ex_ = 320 nm, λ_em_ = 405 nm
ACE	≥2 units/mg, rabbit lung	hippuryl-histidyl-leucine	*o*-phthaldialdehyde	λ_ex_ = 360 nm, λ_em_ = 485 nm

DMPTB: 2,3-Dimercapto-1-propanol tributyrate; DTNB: 5,5′-dithiobis(2-nitrobenzoic acid); FRET: fluorescence resonance energy transfer; λ_ex_: excitation wavelength; λ_em_: emission wavelength.

**Table 2:** Phenolic composition of raw sacha inchi seeds.

Phenolic Compounds	Retention Times (min)	Concentrations (µg/100 g DW)
**Flavonoids**		
Myricetin	37.82–37.91	ND
Quercetin	42.91–43.16	71.65 ± 0.12 ^c^
Luteolin	43.81–43.88	ND
Naringenin	43.86–43.90	ND
Hesperitin	45.27–45.33	ND
Kaempferol	46.20–46.25	ND
Apigenin	46.57–46.64	ND
Isorhamnitin	46.92–46.96	ND
**Phenolic acids**		
Gallic acid	4.82–4.87	ND
4-Hydroxybenzoic acid	11.95–12.06	ND
Chlorogenic acid	13.17–13.30	ND
Vanillic acid	14.43–14.54	ND
Caffeic acid	15.43–15.56	ND
Syringic acid	16.15–16.26	ND
*p*-Coumaric acid	22.32–22.48	84.38 ± 0.43 ^b^
Ferulic acid	25.45–25.60	90.26 ± 0.85 ^a^
Sinapic acid	26.17–26.34	ND

All data are shown as mean ± SD of triplicate experiments. Different lowercase superscripts within the same column demonstrate significant differences at *p* < 0.05, analyzed using an ANOVA and Duncan’s multiple range test. DW: dry weight; ND: not detected.

**Table 3:** Total phenolic contents (TPCs) and antioxidant activities determined via ORAC, FRAP and DPPH radical scavenging assays of raw and roasted sacha inchi seeds.

Sacha Inchi Seeds	TPCs (mg GAE/100 g DW)	Antioxidant Activities (µmol TE/100 g DW)		
		ORAC Assay	FRAP Assay	DPPH Radical Scavenging Assay
Raw	45.15 ± 3.26 ^b^	106.14 ± 9.35 ^d^	2.47 ± 0.41 ^c^	0.012 ± 0.001 ^b^
Roasted-CP	58.13 ± 4.99 ^a^	284.48 ± 14.76 ^a^	4.27 ± 0.30 ^a^	0.015 ± 0.001 ^a^
Roasted-VO	56.03 ± 3.64 ^a^	161.42 ± 14.20 ^c^	3.68 ± 0.49 ^b^	0.014 ± 0.000 ^b^
Roasted-TD	40.83 ± 0.84 ^c^	251.92 ± 17.74 ^b^	4.07 ± 0.88 ^ab^	0.018 ± 0.001 ^a^

All data are shown as mean ± SD of triplicate experiments. Different lowercase superscripts within the same column demonstrate significant differences at *p* < 0.05, analyzed using an ANOVA and Duncan’s multiple range test. Roasted samples were obtained by roasting sacha inchi seeds in a cooking pan (CP), vacuum oven (VO) and hot-air tray dryer (TD). GAE: gallic acid equivalent; DW: dry weight; TE: Trolox equivalent.

**Table 4:** Inhibitory activities of disease-associated enzymes in raw and roasted sacha inchi seeds.

Sacha Inchi Seeds	Inhibitory Activities (% Inhibition)			
	α-Amylase	Lipase	AChE	BchE
Raw	ND	14.63 ± 0.57 ^d^	ND	ND
Roasted-CP	ND	16.82 ± 0.38 ^c^	12.25 ± 1.83	22.48 ± 2.95 ^a^
Roasted-VO	16.33 ± 1.03	24.19 ± 1.60 ^a^	ND	4.46 ± 1.13 ^b^
Roasted-TD	ND	19.50 ± 1.23 ^b^	ND	ND

All data are shown as mean ± SD of triplicate experiments. Different lowercase superscripts within the same column demonstrate significant differences at *p* < 0.05, analyzed using an ANOVA and Duncan’s multiple range test. Roasted samples were obtained by roasting sacha inchi seeds in a cooking pan (CP), vacuum oven (VO), and hot-air tray dryer (TD). The final concentration of all tested extracts was 0.4 mg/mL. AChE: acetylcholinesterase; BChE: butyrylcholinesterase; ND: not detected.

**Table 5:** Inhibition of non-enzymatic glycation reactions of bovine serum albumin (BSA) induced by either methylglyoxal (MG) or D-glucose using raw and roasted sacha inchi seeds.

Sacha Inchi Seeds	Inhibitory Activities (% Inhibition)	
	MG-Induced Glycation	D-Glucose-Induced Glycation
Raw	15.18 ± 1.24 ^c^*	19.40 ± 0.93 ^c^
Roasted-CP	72.66 ± 1.10 ^a^*	75.99 ± 2.05 ^a^
Roasted-VO	67.27 ± 1.98 ^b^	68.55 ± 4.91 ^b^
Roasted-TD	66.80 ± 2.99 ^b^	68.74 ± 1.26 ^b^

All data are shown as mean ± SD of triplicate experiments. Different lowercase superscripts within the same column demonstrate significant differences at *p* < 0.05 analyzed using ANOVA and Duncan’s multiple range test. An asterisk (*) indicates significant difference between the values within the same row at *p* < 0.05, determined using an unpaired *t*-test. Roasted samples were obtained by roasting sacha inchi seeds in a cooking pan (CP), vacuum oven (VO), and hot-air tray dryer (TD). The final concentration of all tested extracts was 0.125 mg/mL.

## Data Availability

Data are contained within this research article and its Supplementary Materials.

## Supplementary Materials

The following supporting information can be downloaded at: https://www.mdpi.com/article/10.3390/foods12224178/s1, Table S1: Proximate composition of raw sacha inchi seeds; Table S2: Moisture contents and color values of raw and roasted sacha inchi seed samples; Figure S1: Physical appearance of raw sacha inchi seeds.
