# Supplementary material for: The Effects of Different Roasting Methods on the Phenolic Contents, Antioxidant Potential, and In Vitro Inhibitory Activities of Sacha Inchi Seeds
Source: Foods. 2023 Nov 20;12(22):4178. doi: 10.3390/foods12224178 (PMC10670140; doi:10.3390/foods12224178)
Supplement: Supplementary file 1 [file foods-12-04178-s001.zip › foods-2680234-supplementary.pdf]

Supplementary Materials

The Effects of Different Roasting Methods on the Phenolic Contents, Antioxidant Potential, and In Vitro Inhibitory Activities of Sacha Inchi Seeds

Suwapat Kittibunchakul [1], Varongsiri Kemsawasd [1], Chatrapa Hudthagosol [2], Promluck Sanporkha [2], Suwimol Sapwarobol [3] and Uthaiwan Suttisansanee [1,*]

[1] Food and Nutrition Academic and Research Cluster, Institute of Nutrition, Mahidol University, Salaya, Phuttamonthon, Nakhon Pathom 73170, Thailand; suwapat.kit@mahidol.ac.th (S.K.); varongsiri.kem@mahidol.ac.th (V.K.); uthaiwan.sut@mahidol.ac.th (U.S.)

[2] Faculty of Public Health, Mahidol University, Ratchathewi, Bangkok 10400, Thailand; chatrapa.hud@mahidol.ac.th (C.H.); promluck.san@mahidol.ac.th (P.S.)

[3] Faculty of Allied Health Sciences, Chulalongkorn University, Pathumwan, Bangkok 10330, Thailand; suwimol.sa@chula.ac.th (S.S.)

[*] Correspondence: uthaiwan.sut@mahidol.ac.th

Table S1. Proximate composition of raw sacha inchi seeds

Nutrients	Amounts per 100 g of fresh matter
Energy (kcal)	559.83 ± 14.82
Moisture (g)	6.79 ± 0.23
Protein (g)	25.54 ± 0.52
Total fat (g)	37.43 ± 1.14
Total carbohydrate (g)	27.95 ± 0.62
Ash (g)	3.14 ± 0.17

The proximate composition was analyzed using the standard protocols of the Association of Official Analytical Chemists (AOAC). All data are shown as mean ± SD of triplicate experiments.

Table S2. Moisture contents and color values of raw and roasted sacha inchi seed samples

Measurements	Sacha inchi seeds			
	Raw	Roasted-CP	Roasted-VO	Roasted-TD
Moisture content (%)	7.04 ± 0.23	0.88 ± 0.04	1.05 ± 0.04	1.30 ± 0.04
L*	56.21 ± 0.12	43.66 ± 0.16	46.49 ± 0.05	50.45 ± 0.26
a*	6.69 ± 0.05	13.80 ± 0.04	11.87 ± 0.00	11.79 ± 0.13
b*	23.27 ± 0.18	24.22 ± 0.03	22.71 ± 0.13	25.19 ± 0.55

The moisture content was determined using a moisture analyzer (Model FD-720, Kett Electric Laboratory, Tokyo, Japan). The color was analyzed using a ColorFlex EZ spectrophotometer (Hunter Associates Laboratory, Reston, VA, USA), and reported in terms of CIELAB units. All data are shown as mean ± SD of triplicate experiments. Roasted samples were obtained by roasting sacha inchi seeds in cooking pan (CP), vacuum oven (VO) and hot-air tray dryer (TD). L*, a*, and b* indicate relative lightness/darkness, redness/greenness and yellowness/blueness of the samples, respectively.

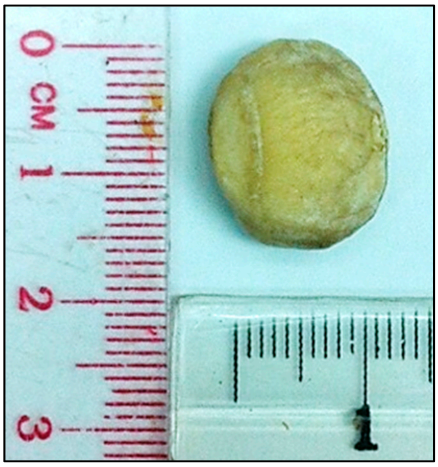

Figure S1. Physical appearance of raw sacha inchi seeds
